# Machine learning-based computer-aided simple triage (CAST) for COVID-19 pneumonia as compared with triage by board-certified chest radiologists

**DOI:** 10.1007/s11604-023-01495-y

**Published:** 2023-10-20

**Authors:** Yoshiharu Ohno, Takatoshi Aoki, Masahiro Endo, Hisanobu Koyama, Hiroshi Moriya, Fumito Okada, Takanori Higashino, Haruka Sato, Noriko Oyama-Manabe, Takafumi Haraguchi, Kazumasa Arakita, Kota Aoyagi, Yoshihiro Ikeda, Shigeo Kaminaga, Akira Taniguchi, Naoki Sugihara

**Affiliations:** 1 Department of Diagnostic Radiology, Fujita Health University School of Medicine, 1-98 Dengakugakubo, Kutsukake-Cho, Toyoake, Aichi 470-1192 Japan; 2 Joint Research Laboratory of Advanced Medical Imaging, Fujita Health University School of Medicine, Toyoake, Aichi Japan; 3 Department of Radiology, University of Occupational and Environmental Health School of Medicine, Kitakyusyu, Fukuoka Japan; 4 Division of Diagnostic Radiology, Shizuoka Cancer Center, Sunto-Gun, Nagaizumi-Cho, Shizuoka Japan; 5 Department of Radiology, Advanced Diagnostic Medical Imaging, Kobe University Graduate School of Medicine, Kobe, Hyogo Japan; 6 Department of Radiology, Ohara General Hospital, Fukushima, Fukushima Japan; 7 Department of Radiology, Oita Prefectural Hospital, Oita, Oita Japan; 8 Department of Radiology, National Hospital Organization Himeji Medical Center, Himeji, Hyogo Japan; 9 Department of Radiology, Oita University Faculty of Medicine, Yufu, Oita Japan; 10 Department of Radiology, Jichi Medical University Saitama Medical Center, Saitama, Saitama Japan; 11 Department of Advanced Biomedical Imaging and Informatics, St. Marianna University School of Medicine, Kawasaki, Kanagawa Japan; 12 Canon Medical Systems Corporation, Otawara, Tochigi Japan

**Keywords:** Lung, Multidetector computed tomography, COVID-19, Decision support systems, Machine learning

## Abstract

**Purpose:**

Several reporting systems have been proposed for providing standardized language and diagnostic categories aiming for expressing the likelihood that lung abnormalities on CT images represent COVID-19. We developed a machine learning (ML)-based CT texture analysis software for simple triage based on the RSNA Expert Consensus Statement system. The purpose of this study was to conduct a multi-center and multi-reader study to determine the capability of ML-based computer-aided simple triage (CAST) software based on RSNA expert consensus statements for diagnosis of COVID-19 pneumonia.

**Methods:**

For this multi-center study, 174 cases who had undergone CT and polymerase chain reaction (PCR) tests for COVID-19 were retrospectively included. Their CT data were then assessed by CAST and consensus from three board-certified chest radiologists, after which all cases were classified as either positive or negative. Diagnostic performance was then compared by McNemar’s test. To determine radiological finding evaluation capability of CAST, three other board-certified chest radiologists assessed CAST results for radiological findings into five criteria. Finally, accuracies of all radiological evaluations were compared by McNemar’s test.

**Results:**

A comparison of diagnosis for COVID-19 pneumonia based on RT-PCR results for cases with COVID-19 pneumonia findings on CT showed no significant difference of diagnostic performance between ML-based CAST software and consensus evaluation (*p* > 0.05). Comparison of agreement on accuracy for all radiological finding evaluations showed that emphysema evaluation accuracy for investigator A (AC = 91.7%) was significantly lower than that for investigators B (100%, *p* = 0.0009) and C (100%, *p* = 0.0009).

**Conclusion:**

This multi-center study shows COVID-19 pneumonia triage by CAST can be considered at least as valid as that by chest expert radiologists and may be capable for playing as useful a complementary role for management of suspected COVID-19 pneumonia patients as well as the RT-PCR test in routine clinical practice.

## Introduction

The new coronavirus disease 2019 (COVID-19) has been spreading worldwide since late 2019 and become a global pandemic involving over 200 countries or regions. Globally, there have been 308 million cumulative cases of COVID-19, with 6.9 million cumulative deaths [1]. COVID-19 is caused by severe acute respiratory syndrome coronavirus-2 (SARS-CoV-2) [2]. The clinical presentation of COVID-19 ranges from asymptomatic or nonspecific mild illness to severe pneumonia with acute respiratory distress syndrome, progression to severe and fatal respiratory failure, and death. Currently, screening or early diagnosis of COVID-19 is one of the key procedures for management of various disease patients suspected of possibly having COVID-19; reverse transcription polymerase chain reaction (RT-PCR) is the gold standard for diagnosing COVID-19. Moreover, some studies also suggest that chest computed tomography (CT) findings in particular may make a positive contribution to deciding that the result of a RT-PCR test is negative [3–6]. In view of these various findings and suggestions, several reporting systems have been recommended for reporting the chest radiographic and CT results of patients who are suspected of having COVID-19 in a high-disease-prevalence setting [7–12]. These systems provide standardized language and diagnostic categories to establish the likelihood that certain lung abnormalities on CT images represent COVID-19, although inter-observer agreements for establishing each category in these reporting systems vary [7–17]. Several studies have therefore used artificial intelligences (AIs) with various approaches for diagnosis of COVID-19 [18–20]. We developed and tested a machine learning (ML)-based CT texture analysis software [21–24]. Moreover, the software has been modified to simplify triage of COVID-19 based on the current RSNA Expert Consensus Statement system and firstly tested in a multi-center and multi-reader study. Moreover, this study was also performed to obtain the official approval for use in a clinical setting from the Japanese Pharmaceuticals and Medical Devices Agency (PMDA).

We hypothesized that ML-based computer-aided simple triage (CAST) software could be as sensitive as board-certified chest radiologists with good acceptance rates for radiological finding classification of lung parenchyma and could function as a substitute for radiologists for diagnosing patients with suspected COVID-19. The purpose of this study was, thus, to determine the capability of ML-based CAST software based on RSNA expert consensus statements to diagnose COVID-19 pneumonia in a multi-center and multi-reader study.

## Materials and methods

### Protocol, support, and funding

Training and validation cases were retrospectively obtained with institutional review board approval from Fujita Health University Hospital and Kansai Medical University Medical Center. Both institutions received research grants from Canon Medical Systems.

The test study was a retrospective study approved by the certified review board of Fujita Health University Hospital, which served as the coordinating center, and subsequently approved by the directors of Jichi Medical University Saitama Medical Center and St. Marianna University School of Medicine prior to site initiation. This study was compliant with the Health Insurance Portability and Accountability Act, written informed consent was waived, and it was technically and financially supported by Canon Medical Systems Corporation. Moreover, this study was financially supported by Grants-in-Aid for Scientific Research from the Japanese Ministry of Education, Culture, Sports, Science and Technology (JSTS.KAKEN; No. 18K07675 and JSTS.KAKEN; No. 20K08037) and the Smoking-Research Foundation.  Six of the authors, K.A., Ko. Ao., Y.I., S.K., A.T. and N.S., are employees of Canon Medical Systems Corporation (Otawara, Tochigi, Japan) who did not have control over any of the data used in this study. Two employees of Micron, Inc., (Tokyo, Japan), which is an international clinical research organization (CRO), handled all data, and performed all statistical analyses independently from the authors.

### Subjects

#### Training and validation cases

As the training set for this study, 656 cases obtained between March 2020 and March 2021 from Fujita Health University and Kansai Medical University Medical Center were included. These cases comprised 384 males (55 ± 22 years, age range 10–94 years) and 272 females (53 ± 22 years, age range 11–96 years) with COVID-19 pneumonia (*n* = 191) and non-COVID-19 pneumonia (*n* = 465). Another 137 cases were selected as the validation set from Fujita Health University by applying the same inclusion criteria as for selecting the test cases. For the validation set, 137 cases were selected, consisting of 62 males (50 ± 20 years, age range 15–85 years) and 75 females (49 ± 20 years, age range 18–85 years) with COVID-19 pneumonia (*n* = 28) and non-COVID-19 pneumonia (*n* = 109).

#### Test cases

The patients originally included were recruited between January 2020 and June 2021 at Jichi Medical University Saitama Medical Center (institution A) and St. Marianna University School of Medicine (institution B). Their radiologists did not perform any image studies or statistical analyses for this study. Institution A was placed in the prefecture without collapse of the medical system, and institution B was placed in the prefecture with collapse of the medical system. The inclusion criteria for the trial were patients (1) with suspected COVID-19 infection whose chest CT and RT-PCR for SARS-CoV-2 had been obtained from a pharyngeal or nasopharyngeal swab specimen, (2) whose RT-PCR examinations for SARS-CoV-2 were performed and data collected within 6 days from chest CT examination, and (3) whose initial chest CT data had been obtained from an unenhanced CT of the entire lung performed with a CT system provided by Canon Medical Systems and reconstructed with a section thickness and gap equal to or less than 1.0mm and a high frequency algorithm, and using two lung kernels (FC51 and FC52, Canon Medical Systems) by means of filter back projection (FBP) or hybrid-type iterative reconstruction (adaptive dose reduction using 3D processing [AIDR 3D], Canon Medical Systems). The exclusion criteria were patients (1) who had undergone a lobectomy, (2) who had suffered lobar atelectasis, (3) with intubation of the trachea, (4) whose CT examination results showed severe artifacts due to body movement, (5) without any data sheets, and (6) who refused to be included in this study.

**Fig. 1:**
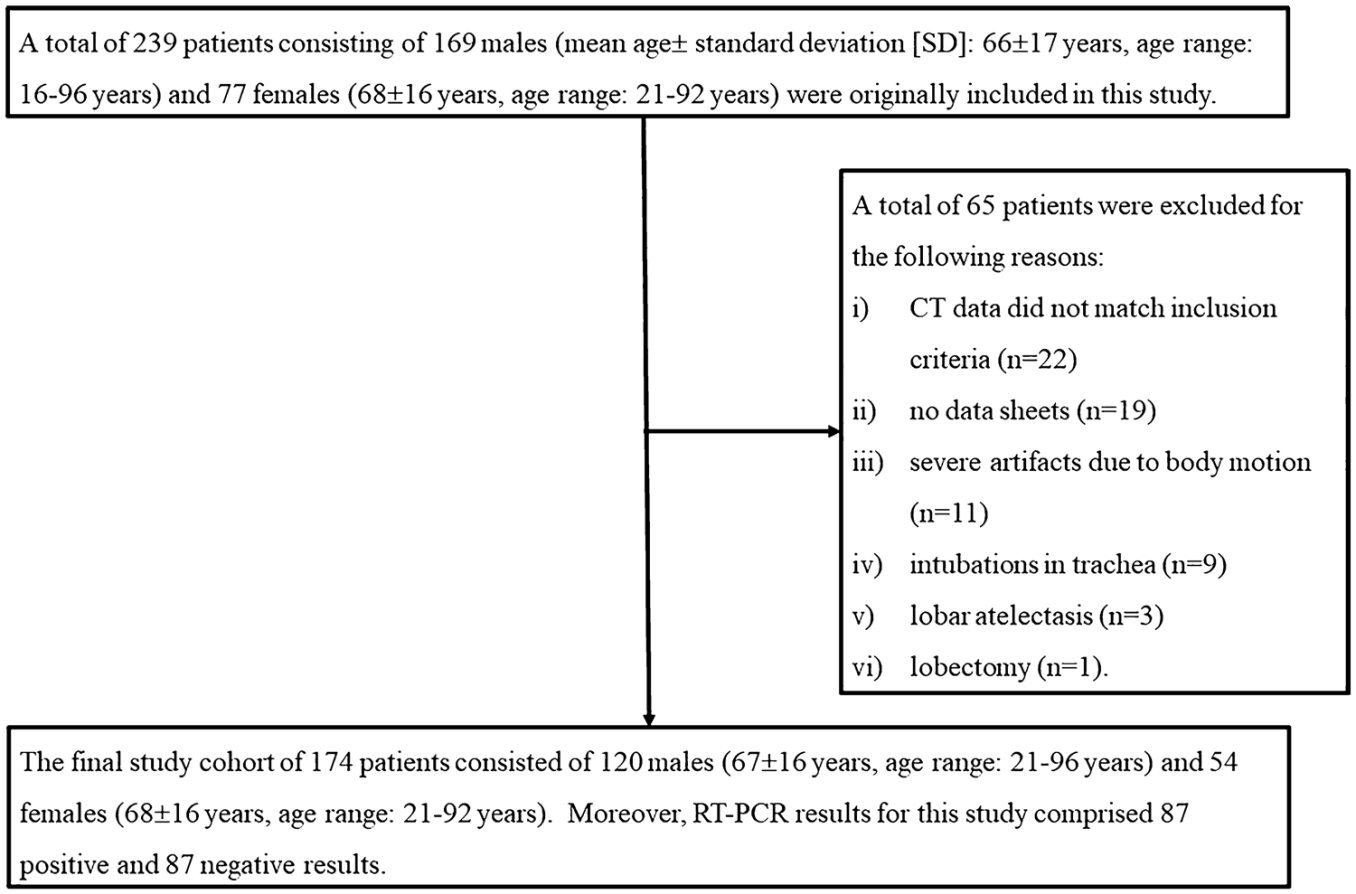
Patient flow chart. A total of 239 patients were originally included in this study, and 65 patients were excluded for the reasons detailed in the figure, so that eventually 174 patients were included in this study

Of the total of 239 patients consisting of 162 males (66 ± 17 years, age range 16–96 years) and 77 females (68 ± 16 years, age range 21–92 years) originally included in this study, 65 were excluded due to (i) CT data not matching with inclusion criteria (*n* = 22), (ii) no data sheets (*n* = 19), (iii) severe artifacts due to body motion (*n* = 11), (iv) intubation of the trachea (*n* = 9), (v) lobar atelectasis (*n* = 3), and (vi) lobectomy (*n* = 1). The final study cohort comprised 174 patients consisting of 120 males (67 ± 16 years, age range 21–96 years) and 54 females (68 ± 16 years, age range 21–92 years), and 87 positive and 87 negative RT-PCR results. In this study, 87 non-COVID-19 cases consisted of cardiac, liver, or renal dysfunction (*n* = 37), malignant tumor with and without lung metastasis or lymphangitis carcinomatosis (*n* = 20), other infectious diseases (*n* = 18) due to bacterial (*n* = 7) and viral (*n* = 5) infections, mycobacterial infection (*n* = 4), and pulmonary tuberculosis (*n* = 2), interstitial lung disease (*n* = 7), organizing pneumonia (*n* = 3), and asthma (*n* = 2). The flow chart for patient selection is shown in Fig. 1, and details of patient characteristics are listed in Table 1.

**Table 1:** Patient characteristics and statistical differences between two institutions with and without collapse of medical system

		Total study cohort (*n* = 174)	Subgroup at each institution		*p* value
			Institution A (*n* = 90)	Institution B (*n* = 84)	
Age (years)	(Mean ± SD)	67.0 ± 15.9 (21–96)	63.5 ± 15.7 (21–86)	70.7 ± 15.2 (33–96)	0.001
Gender	Male: female	120: 54	57: 33	66: 22	0.05
Height (cm)	(Mean ± SD)	163.3 ± 10.4	162.8 ± 10.5	163.9 ± 10.2	0.25
Body weight (kg)	(Mean ± SD)	62.1 ± 18.1	63.0 ± 16.3	60.9 ± 20.3	0.24
Clinical symptoms	Number (%)	141 (81.0%)	61 (67.8%)	80 (95.2%)	< 0.0001
Fever	Number (%)	132 (75.9%)	58 (64.4%)	74 (88.1%)	0.0004
Time between onset of clinical symptoms and CT examination (days)	(Mean ± SD)	3.9 ± 4.6	0.60 ± 1.18	4.6 ± 4.7	< 0.0001
Time between CT and RT-PCR for SARS-CoV-2 examinations (days)	(Mean ± SD)	0.8 ± 1.9 (− 5–6)	1.5 ± 2.4 (− 5–6)	0.1 ± 1.0 (− 1–6)	< 0.0001
RT-PCR results	Positive: negative	87: 87	47: 43	40: 44	0.65
Presence vs. absence of COVID-19 pneumonia on CT	Presence: absence	95: 79	41: 49	54: 30	0.02
Positive appearance vs. atypical appearance or negative for pneumonia on CT for COVID-19 pneumonia	Positive: atypical or negative	41: 133	14: 76	27: 57	0.01
CT disease severity score	(Mean ± SD)	8.4 ± 6.6 (0–23)	5.1 ± 5.6 (0–20)	11.9 ± 5.9 (0–23)	< 0.0001
CTDIvol (mGy)	(Mean ± SD)	14.4 ± 9.1 (3.4–35.3)	19.5 ± 9.4 (4.8–35.3)	8.9 ± 4.3 (3.4–29.3)	< 0.0001
DLP (mGy cm)	(Mean ± SD)	502.7 ± 318.2 (113.7–1482.6)	672.2 ± 335.0 (166.4–1482.6)	321.2 ± 162.1 (113.7–1148.6)	< 0.0001
Effective dose(mSv)	(Mean ± SD)	7.0 ± 4.5 (1.6–20.8)	9.4 ± 4.7 (2.3–20.8)	4.5 ± 2.3 (1.6–16.1)	< 0.0001

*SD* standard deviation, *COVID-19* coronavirus disease 2019, *SARS-CoV-2* severe acute respiratory syndrome coronavirus-2, *RT-PCR* reverse transcription polymerase chain reaction, *CTDIvol* volume computed tomography dose index, *DLP* dose length product

### CT examinations

The CT data were obtained with two 80-, one 160- and one 320-detector row CT scanners (Aquilion PRIME, Aquilion Precision and Aquilion ONE; Canon Medical Systems, Otawara, Tochigi, Japan). The following numbers of patients were scanned with one of the CT systems: 80-detector row CT, 649; 160-detector row CT, 3; and 320-detector row CT, 4. CT examinations were performed with unenhanced CT with helical scanning by using the following parameters: 64–80 × 0.5 mm collimation, auto mA with image standard deviation (SD) ranged between 8 and 13, 120 kVp, 0.813–0.891 beam pitch, 0.35–0.5 s gantry rotation time, 512 × 512 matrix, and 234–410 mm field of view. All thin-section CT data were then reconstructed with filtered back projection or hybrid iterative reconstruction (AIDR 3D: Canon Medical) method in contiguous section thicknesses of 0.5 mm or 1 mm and then used for generating the lung reconstruction kernel as FC51 or FC52 (Canon Medical). The estimated volume computed tomography dose index (CTDI_vol_) displayed on the CT scanner console was recorded for each patient. These values were based on the weighted computed tomography dose index (CTDI_w_ [e.g., tube voltage or tube current]). CTDI_vol_ obtained in this study was assessed as 10.63 ± 5.22 (mean ± SD) mGy and ranged from 2.7 to 33.6 mGy. The estimated dose length product (DLP) was calculated as CTDI_vol_ × scan length, which was determined as 91–1900 mGy × cm, with the effective dose for this protocol estimated at 1.27–26.60 mSv. All CT examinations were performed with breath holding at full inspiration.

For the test cases, all CT data were obtained with two 64-, one 80-, and two 320-detector row CT scanners (Aquilion 64, Aquilion PRIME and Aquilion ONE, respectively; Canon Medical Systems, Otawara, Tochigi, Japan). Sixty-nine patients were scanned with the 64-detector row CT, 84 with the 80-detector row CT, and 21 with the 320-detector row CT. CT examinations were performed with unenhanced CTs with helical scanning using the following parameters: 64–80 × 0.5 mm collimation, auto mA with image standard definition (SD) ranged between 7 and 15, 120 kVp, 0.81–0.89 beam pitch, 0.35–0.5 s gantry rotation time, 512 × 512 matrix and 320–500 mm field of view. All thin-section CT data were then reconstructed with filtered back projection or hybrid iterative reconstruction (AIDR 3D: Canon Medical) in contiguous section thicknesses of 0.5 mm or 1 mm and used for generating the lung reconstruction kernels as FC51 or FC52 (Canon Medical). The estimated volume computed tomography dose index (CTDI_vol_) displayed on the CT scanner console was recorded for each patient. These values were based on the weighted computed tomography dose index (CTDI_w_ [e.g., tube voltage or tube current]). CTDI_vol_ obtained in this study was assessed as 14.4 ± 9.1 (mean ± SD) mGy and ranged from 3.4 to 35.3 mGy. The estimated dose length product (DLP) was calculated as CTDI_vol_ × scan length, which was determined as 113.7–1482.6 mGy × cm, with the effective dose for this protocol estimated at 1.6–20.8 mSv. All CT examinations were performed with breath holding at full inspiration. Details of the unenhanced CT protocol can be found in Table 2.

**Table 2:** CT protocols for multi-center and multi-reader studies

Unenhanced CT protocol			
CT system	64-detector row CT (Aquilion 64: Canon Medical Systems)	80-detector row CT (Aquilion PRIME: Canon Medical Systems)	320-detector row CT (Aquilion ONE: Canon Medical Systems)
Scan mode	Helical		
Detector collimation	0.5×64	0.5×80	0.5×64
Tube current (mA)	Automatic exposure control		
Image SD for automatic exposure control	12.0 ± 0.2 (10.0–12.0)	7.9 ± 1.7 (7.0–15.0)	14.6 ± 1.7 (7.0–15.0)
Tube voltage (kVp)	120		
Beam pitch	0.813–0.891	0.813	0.813
Gantry speed (s/rotation)	0.5	0.35–0.5	0.5
Section thickness (mm)	0.5 or 1.0		
FOV (mm)	300–380	320–400	300–380
Matrix	512×512		
Reconstruction method and kernel	Filter back projection (FBP) or hybrid-type IR (AIDR 3D: Canon Medical Systems) and high frequency kernel (FC51 or FC52: Canon Medical Systems)		
Radiation dose (CTDI_vol_: mean ± SD mGy) (range)	22.9 ± 7.9 (5.7—35.3)	9.0 ± 4.3 (3.4—29.3)	7.9 ± 2.7 (4.8—15.8)
Dose length product (DLP: mean ± SD mGy cm) (range)	787.4 ± 292.9 (196.7—1482.6)	324.1 ± 163.5 (113.7—1148.6)	282.0 ± 93.2 (166.4—545.1)
Effective dose (mean ± SD mSv) (range)	11.0 ± 4.1 (2.8—20.8)	4.5 ± 2.3 (1.6—16.1)	3.9 ± 1.3 (2.3—7.6)

*SD* Standard deviation, *FOV* Field of view, *CTDI*_*vol*_ Estimated volume computed tomography dose index, *DLP* Dose length product

### Machine-learning CAST software

**Fig. 2:**
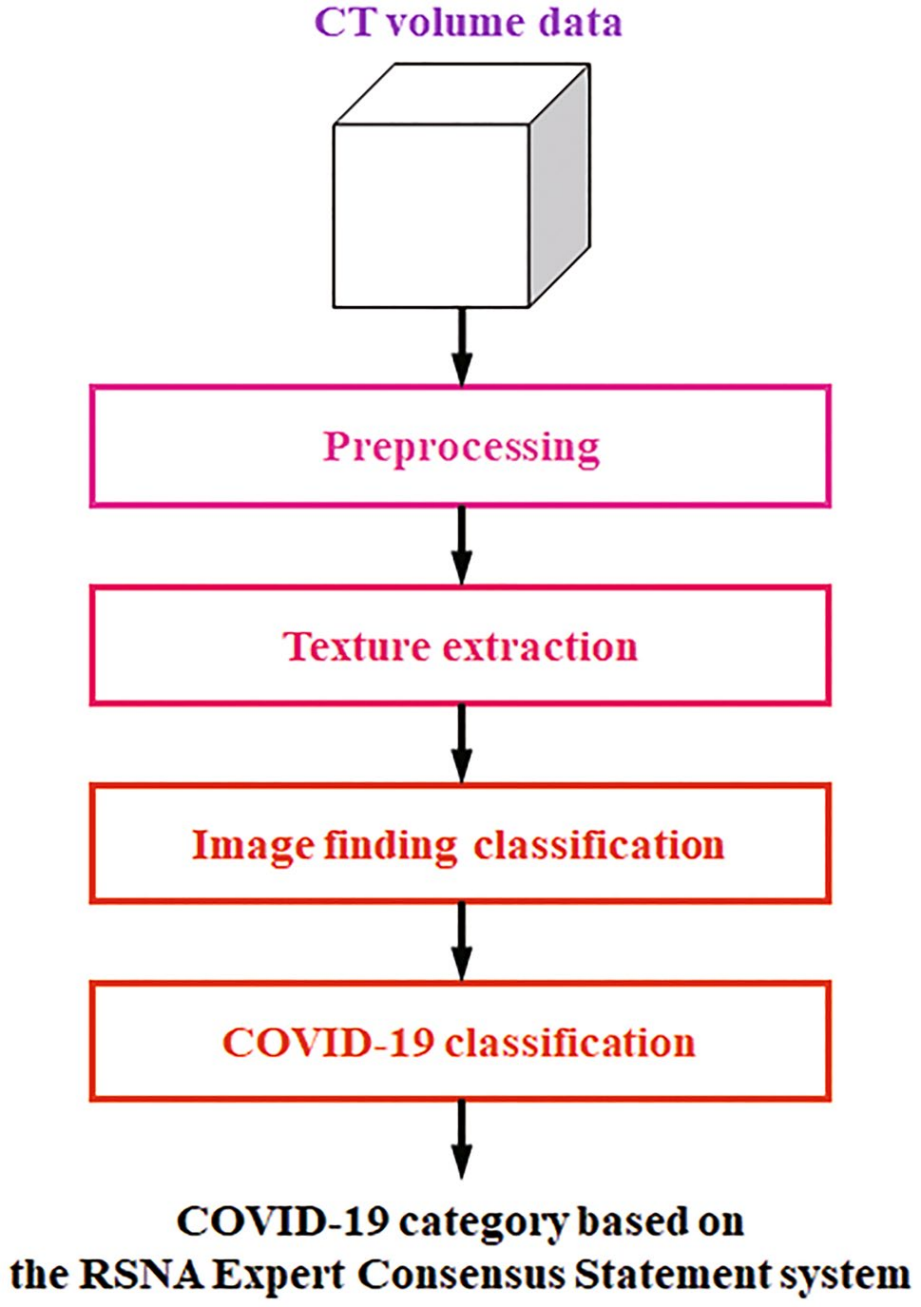
Flow chart for machine-learning-based CAST software. When given a chest CT volume datum as input, the software segments the lung region and the lung lobes automatically in the processing stage. The resultant lung and lobe masks are then used in the subsequent texture extraction and image findings classification stages. In the texture extraction stage, the likelihood of occurrence of one of seven texture patterns is calculated for every single voxel: (1) normal lung, (2) ground-glass opacity, (3) reticulation, (4) emphysema, (5) nodular lesion, (6) consolidation, and (7) honeycombing. The multi-class support vector machine is then used to calculate the probability of occurrence of each texture pattern for each voxel. Finally, each voxel is labeled with a specific texture pattern with the highest probability of occurrence. In the image finding classification stage, the typical image findings information defined in the RSNA COVID-19 report format is determined by an image analysis algorithm. This algorithm identifies the quantity of GGO and its morphology, as well as reticulation, consolidation, nodular lesion, anatomical location and the positional relationship among each of the textures to identify the typical image findings for COVID-19. The COVID-19 classification algorithm is based on the Random Forest machine learning model for classification of the probability of COVID-19 pneumonia occurrence using the findings obtained in the previous stage. This algorithm is used to classify each CT data set into four patterns: (1) typical (2) indeterminate, (3) atypical, and (4) negative for pneumonia

Figure 2 shows the flow chart of the ML-based software for CAST. Given the chest CT volume data as input, it classifies each voxel into seven radiological texture patterns. This process is called texture extraction. The results of the texture extraction are then used to determine ten radiological findings related to COVID-19 pneumonia as described in the RSNA Expert Consensus Statement system [7, 8]. This process is known as image findings classification. Finally, image appearance is classified into four categories. This categorization yields the final classification of positive and negative for COVID-19 pneumonia.

#### Preprocessing

Given chest CT volume data as input, the software automatically segments the lung region and the lung lobes. The resultant lung and lobe masks are then used in the subsequent texture extraction and image findings classification.

#### Texture extraction

For this stage, the likelihood of occurrence of one of seven texture patterns is calculated for every single voxel: (1) normal lung, (2) ground-glass opacity, (3) reticulation, (4) emphysema, (5) nodular lesion, (6) consolidation, and (7) honeycombing. The extremely randomized trees method is used to calculate the likelihood of occurrence of all the textures except nodular lesion, for which the radial structured tensor method is used. The multi-class support vector machine is then used to calculate the probability of occurrence of each texture pattern for every voxel. Finally, each voxel is labeled with a specific texture pattern with the highest probability of occurrence. Details of the texture extraction algorithm can be found in the literature [22–24].

#### Image findings classification

This step calculates the typical image findings defined in the RSNA COVID-19 report format and determined by an image analysis algorithm. This texture information for each voxel obtained in the previous step is then used to calculate the image findings information. In particular, the algorithm identifies the quantity of GGO and its morphology, as well as reticulation, consolidation, nodular lesion, anatomical location, and the positional relationship among each of the texture to identify the typical image findings for COVID-19.

#### COVID-19 classification

The image analysis algorithm is used for COVID-19 pneumonia imaging classification according to the RSNA classification system for COVID-19 pneumonia into four patterns: (1) typical (2) indeterminate, (3) atypical, and (4) negative for pneumonia [7, 8]. The algorithm is based on the Random Forest machine learning model for classification of the probability of COVID-19 pneumonia occurrence using the findings obtained in the previous step.

### Image analysis

All CAST evaluations with ML-based CAST software were performed on a workstation (Vitrea, Canon Medical Systems). All qualitative image analyses were performed on an image reading system (IRUMneo Report, Micron, Inc., Tokyo, Japan). All investigators involved in this study reviewed all CT data without having access to any information about clinical symptoms, RT-PCR data or results of ML-based CT texture analysis and CAST.

#### Diagnosis of COVID-19 pneumonia and subtype classification based on the RSNA expert consensus statement system performed with ML-based CAST software, by consensus evaluation, and by each investigator

For diagnosis of COVID-19 pneumonia and subtype classification for each patient based on the RSNA expert consensus statement system, three board-certified chest radiologists (M.E., H.K., and H.M.) with 20-, 31-, and 40-year experience, respectively, reviewed data obtained with unenhanced CT with the level of the lung window set at -550HU and the width at 1600HU. First, the three investigators evaluated each CT data set based on the RSNA expert consensus statement system into three categories: (1) positive (typical or indeterminate), (2) atypical, and (3) negative cases. Second, diagnosis of COVID-19 pneumonia in each case was assessed as positive or negative (atypical or negative). Third, a qualitative CT severity scoring method introduced by Pan et al. [25] was used to calculate the extent of anatomic involvement for each of the 5 lobes as: 0, no involvement; 1, < 5% involvement; 2, 5–25% involvement; 3, 26–50% involvement; 4, 51–75% involvement; and 5, > 75% involvement. The resultant global CT score was then calculated by summing the individual lobar scores with a possible range of a minimum of 0 to a maximum of 25. In each case, the final category based on the RSNA expert consensus statement and diagnosis of COVID-19 pneumonia was established by majority agreement among the three investigators. When a case was assessed as a different category by each of the investigators and none of them could determine each final evaluation based on majority category in some cases, another board-certified chest radiologist (T.A.) with a 32-year experience, who performed as a central reviewer for this study, assessed the final category without any information about clinical symptoms, RT-PCR data, results of ML-based CT texture analysis, ML-based CAST software or the three investigators’ evaluation results for these cases. Moreover, the final qualitative CT severity score in each case was determined as the average of the values obtained from the three investigators.

#### Agreements for CT texture analysis of ML-based CAST software and three other investigators

For determination of agreement between findings obtained with ML-based CT texture analysis and by three other board-certified chest radiologists (T.H., F.O. and H.S.) with 8-, 25-, and 33-year experience, respectively, the same board-certified chest radiologist who acted as a central reviewer selected 305 slices with 6 different lung structures from 196 cases based on the glossary of the Fleischner Society [21], namely (i) consolidation, (ii) emphysema, (iii) ground-glass opacity (GGO), (iv) honeycombing, (v) nodular lesion, and (vi) reticulation. From the 305 slices, 156 were randomly selected to determine agreements between findings obtained with ML-based CT texture analysis and by the three investigators. Without any information about results determined by the central reviewer, the three chest radiologists then assessed the results for ML-based CT texture analysis of each radiological finding with the following 5-point scoring system: (1) true positive and agreement on ML-based CT texture analysis results for a targeted structure (i.e., analysis of results for a targeted area within the ROI as more than 80%), (2) true positive and agreement on ML-based CT texture analysis results for a targeted structure (i.e., analysis of results for a targeted area as more than 60% and equal to or less than 80% within ROI), (3) true positive but disagreement on ML-based CT texture analysis results for a targeted structure (i.e., displayed analysis results for targeted area within the ROI as equal to or less than 60%), (4) true negative and agreement on ML-based texture analysis results for a targeted structure, and (5) false positive and disagreement on ML-based texture analysis results for a targeted structure.

### Statistical analysis

To determine the influence of collapse of the medical system on the results of this study, characteristics of patients from the two institutions in the test cohort were compared by two-tailed t-test or Wilcoxon’s signed rank test.

Agreements between findings obtained with CAST software and by each investigator or between those by each investigator for all cases and for cases provided by either institution, as well as inter-observer agreements between those obtained with CAST software and by each investigator, were determined by using Cohen’s kappa statistics with *χ*^2^ test, and inter-rater agreement among all investigators by means of Fleiss’ kappa statistics.

For comparison of diagnosis for COVID-19 pneumonia of all cases based on RT-PCR between ML-based CAST software and consensus evaluation or individual investigator’s evaluations, as well as of all cases with COVID-19 pneumonia findings on CT and cases provided by each institution, sensitivity, specificity, and accuracy of the diagnoses were compared by McNemar’s test.

To determine agreement for each radiological finding evaluation of all slices between ML-based CT texture analysis performed with CAST software and by each investigator, inter-rater agreement among all investigators was evaluated by means of Fleiss’ kappa statistics.

For comparison of the accuracy for each radiological finding obtained with the ML-based CT texture analysis on CAST software and by each investigator, the number of true-positive ROIs was divided by all ROIs. Accuracy for each lung radiological finding evaluation was then compared using McNemar’s test.

All Cohen’s and Fleiss’s kappa statistics were assessed based on past literatures [26–29]. For all Cohen’s kappa statistics, all inter-observer agreements were rated as no agreement for *κ* = 0, slight for 0 < *κ* < 0.21, fair for *κ* = 0.21–0.40, moderate for *κ* = 0.41–0.60, substantial for *κ* = 0.61–0.80, and almost perfect for *κ* = 0.81–1.00 [26]. For all Fleiss’s kappa statistics, all inter-rater agreements were rated as no agreement for *κ* < 0, slight for *κ* = 0.01–0.20, fair for *κ* = 0.21–0.40, moderate for *κ* = 0.41–0.60, substantial for *κ* = 0.61–0.80, and almost perfect for *κ* = 0.81–1.00 [27–29].

For all statistical analyses using commercially available software (JMP 14: SAS Institute Japan, Co. Ltd., Tokyo, Japan; StatMate III: Atoms Co. Ltd., Tokyo, Japan; R: R Foundation for Statistical Computing, Vienna, Austria; and EZR: Saitama Medical Center, Jichi Medical University, Saitama, Japan), a *p* value less than 0.05 was considered statistically significant.

## Results

Patient characteristics and statistical differences between the two institutions with and without collapse of the medical system are shown in Table 1. There were significant differences between the two institutions’ patients in terms of age (*p* = 0.001), clinical symptoms (*p* < 0.0001), fever (*p* = 0.0004), time between onset of clinical symptoms and CT examination (*p* < 0.0001), time between CT and RT-PCR examinations (*p* < 0.0001), presence vs. absence of COVID-19 pneumonia on CT (*p* = 0.02), and positive appearance vs. atypical appearance or negative findings for COVID-19 pneumonia on CT (*p* = 0.01), CT disease severity (*p* < 0.0001), CTDI_vol_ (*p* < 0.0001), DLP (*p* < 0.0001) and effective dose (*p* < 0.0001).

**Fig. 3:**
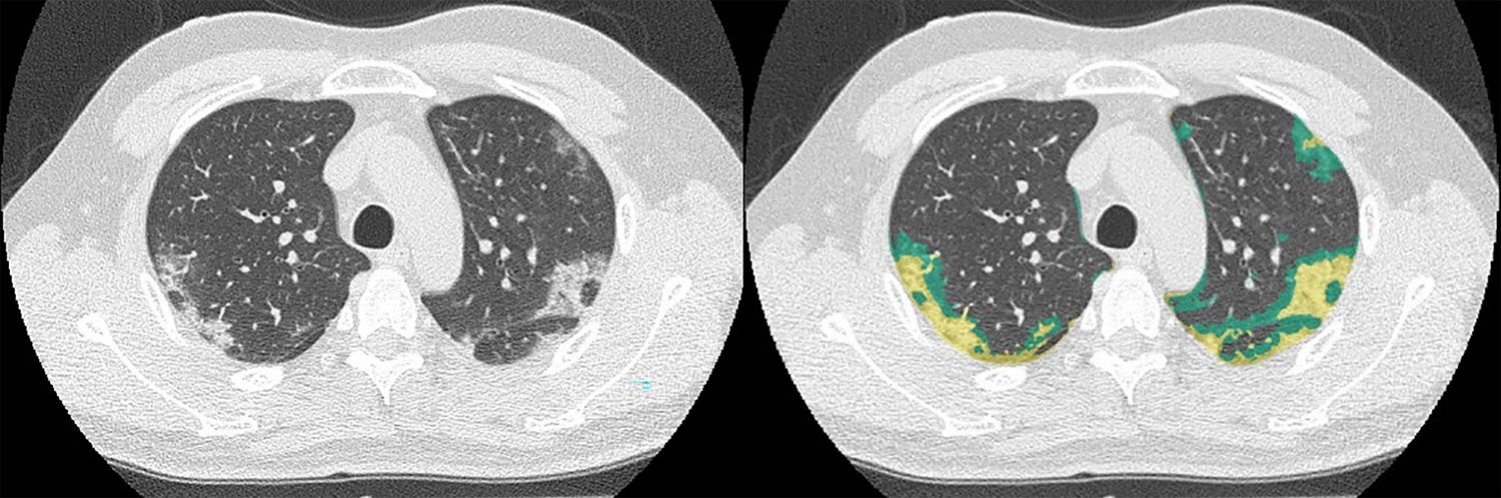
46-year-old male patient diagnosed as COVID-19 pneumonia and as positive on RT-PCR. (L to R: thin-section CT to CAST result) On thin-section CT, ground-glass opacities and reticulation classified as crazy-paving pattern were observed in the peripheral lung in both lungs. CAST shows ground-glass opacities as green and reticulation classified as crazy-paving pattern as yellow in both lungs. The PCR test also identified this case as “positive”. All chest radiologists and the CAST software accurately evaluated this case as “positive case”, and it was therefore classified as true-positive in this study

**Fig. 4:**
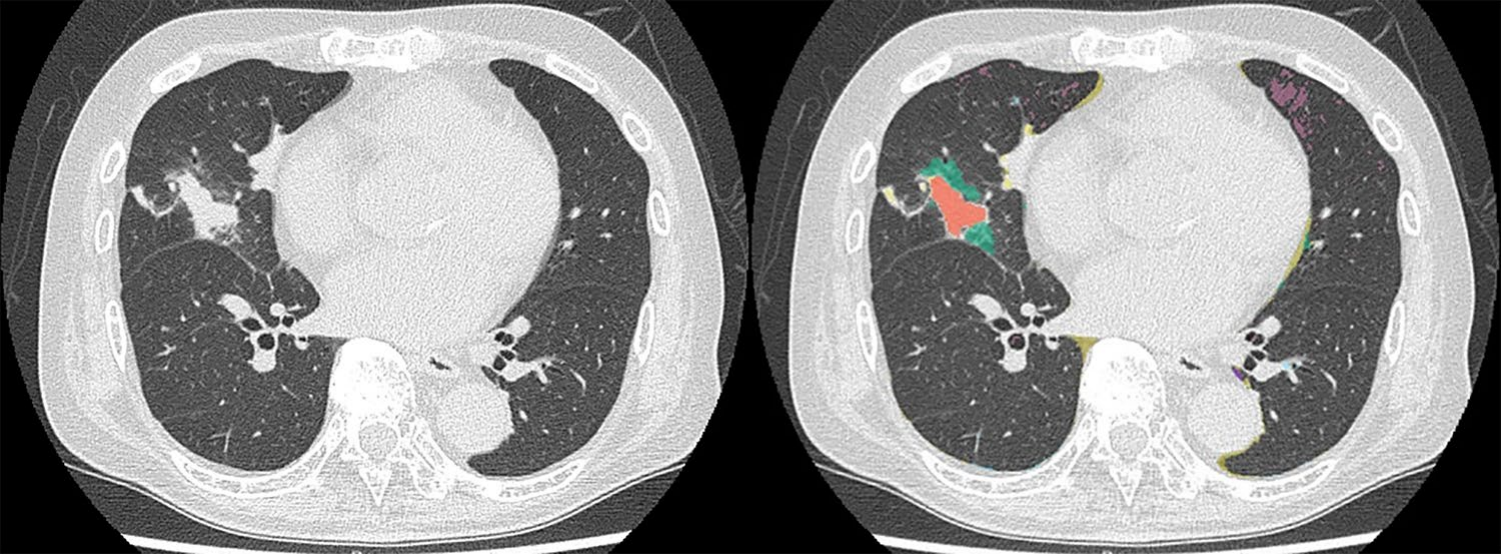
74-year-old male patient with suspected COVID-19 pneumonia and diagnosed as negative on RT-PCR. (L to R: thin-section CT to CAST result) On thin-section CT, consolidation with ground-glass opacities was observed in the right middle lobe, and emphysema in the lingula segment. CAST shows consolidation and ground-glass opacities as pale beige and green in the right middle lobe and emphysema as purple. The PCR test also identified this case as “negative”. All chest radiologists and the CAST software assessed this case as “atypical appearance” and “negative case”, so that it was judged to be true-negative

**Fig. 5:**
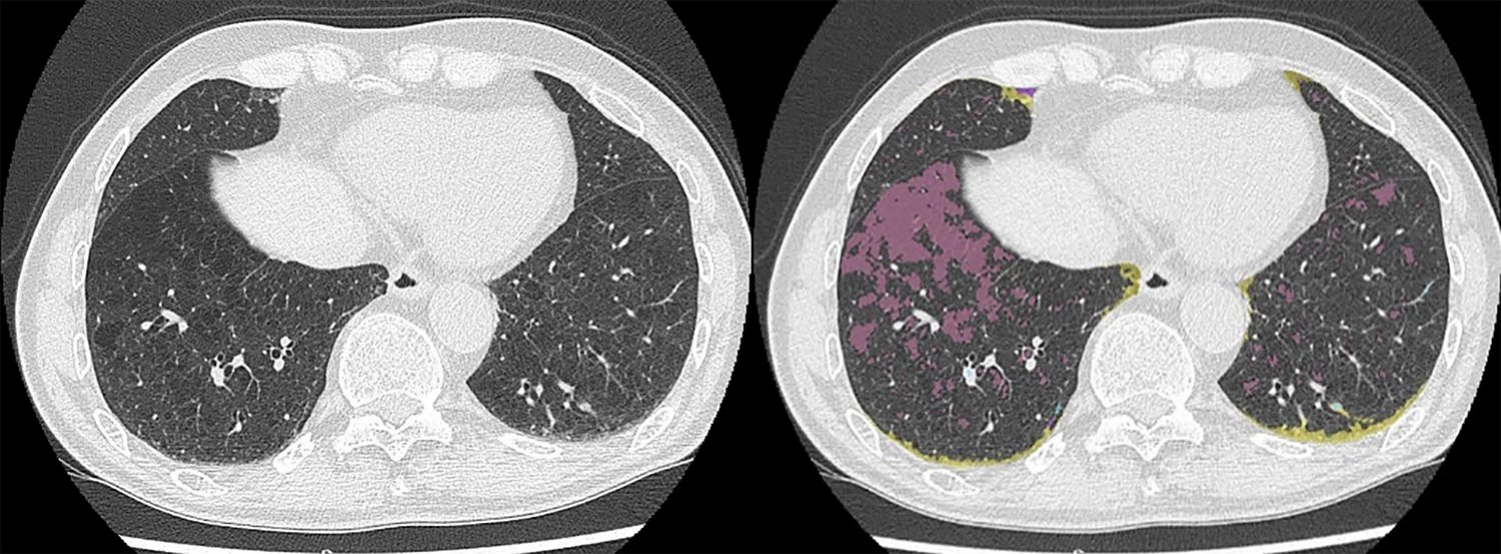
73-year-old male patient with suspected COVID-19 pneumonia and diagnosed as negative on RT-PCR. (L to R: thin-section CT to CAST result) On thin-section CT, no ground-glass opacity or consolidation was observed in either lung. Low attenuation areas assessed as emphysema were observed in both lungs, and reticulations within the peripheral zone in both lungs. CAST shows emphysema as purple and reticulation as yellow. The PCR test also showed this case as “negative”. All chest radiologists and the CAST software assessed this case as “negative for pneumonia” and “negative case”, so that this case was judged to be true-negative

Representative cases are shown in Figs. 3, 4, and 5.

Agreements for diagnosis of COVID-19 pneumonia between CAST software and consensus evaluation or between each investigator’s evaluation of all cases and of cases provided by each institution are shown in Table 3. All agreements for overall cases between CAST software and other means of evaluation were rated as “moderate” (0.42 ≤ *κ* ≤ 0.55, *p* < 0.0001). For cases from institution A, all agreements were rated as significantly “moderate” or “substitution” (0.59 ≤ *κ* ≤ 0.76, *p* < 0.0001), although agreements at institution B for investigator B (*κ* = 0.3, *p* = 0.003) and C (*κ* = 0.19, *p* = 0.01) were rated as significantly “fair” or “slight”. When inter-rater agreements for diagnosis of COVID-19 pneumonia among the three investigators were compared, they were rated as “moderate” (Fleiss’s kappa value = 0.53, *z* = 16.5, *p* < 0.0001).

**Table 3:** Agreements for diagnosis of COVID-19 pneumonia between CAST software and consensus evaluation or evaluation by each investigator of all cases and all cases provided by each institution

	Interobserver agreement	Cohen’s kappa value	*p* value
Overall cases	ML-based Software vs. Final evaluation	0.55	< 0.0001
	ML-based Software vs. Investigator A	0.42	< 0.0001
	ML-based Software vs. Investigator B	0.53	< 0.0001
	ML-based Software vs. Investigator C	0.5	< 0.0001
Institution A cases	ML-based Software vs. Final evaluation	0.76	< 0.0001
	ML-based Software vs. Investigator A	0.65	< 0.0001
	ML-based Software vs. Investigator B	0.59	< 0.0001
	ML-based Software vs. Investigator C	0.71	< 0.0001
Institution B cases	ML-based Software vs. Final evaluation	0.19	0.02
	ML-based Software vs. Investigator A	0.12	0.05
	ML-based Software vs. Investigator B	0.3	0.003
	ML-based Software vs. Investigator C	0.19	0.01

Results of a comparison of diagnosis for COVID-19 pneumonia based on RT-PCR result between those obtained with ML-based CAST software and consensus evaluation or each investigator’s evaluation of all cases and cases with COVID-19 pneumonia findings on CT are shown in Tables 4 and 5.

**Table 4:** Comparison of diagnosis for COVID-19 pneumonia based on RT-PCR results for all cases between ML-based CAST software and consensus evaluation or individual investigators’ evaluations

Groups	Method	SE (%)	SP (%)	AC (%)	p value for SE	p value for SP	p value for AC
Overall cases	ML-based software for CAST	88.5	47.1	67.8	–	–	–
		(77/87)	(41/87)	(118/174)			
	Final investigators. evaluation	88.5	79.3	83.9	1	< 0.0001	< 0.0001
		(77/87)	(69/87)	(146/174)			
	Investigator A’s evaluation	80.5	90.8	85.6	0.07	< 0.0001	< 0.0001
		(70/87)	(79/87)	(149/174)			
	Investigator B’s evaluation	93.1	48.3	70.7	0.22	1	0.49
		(81/87)	(42/87)	(123/174)			
	Investigator C’s evaluation	88.5	85.1	86.8	1	< 0.0001	< 0.0001
		(77/87)	(74/87)	(151/174)			
Institution A	ML-based software for CAST	85.1	76.7	81.1	–	–	–
		(40/47)	(33/43)	(73/90)			
	Final investigators’ evaluation	83	95.3	88.9	1	0.01	0.07
		(39/47)	(41/43)	(80/90)			
	Investigator A’s evaluation	76.6	100	87.8	0.22	0.004	0.21
		(36/47)	(43/43)	(79/90)			
	Investigator B’s evaluation	89.4	67.4	78.9	0.62	0.42	0.81
		(42/47)	(29/43)	(71/90)			
	Investigator C’s evaluation	83	95.3	88.9	1	0.01	0.1
		(39/47)	(41/43)	(80/90)			
Institution B	ML-based software for CAST	92.5	18.2	53.6	–	–	–
		(37/40)	(8/44)	(45/84)			
	Final investigators’ evaluation	95	63.6	78.6	1	0.0002	0.0001
		(38/40)	(28/44)	(66/84)			
	Investigator A’s evaluation	85	81.8	83.3	0.37	< 0.0001	0.0001
		(34/40)	(36/44)	(70/84)			
	Investigator B’s evaluation	97.5	29.5	61.9	0.48	0.27	0.12
		(39/40)	(13/44)	(52/84)			
	Investigator C’s evaluation	95	75	84.5	1	< 0.0001	< 0.0001
		(38/40)	(33/44)	(71/84)			

*SE* sensitivity, *SP* specificity, *AC* accuracy

**Table 5:** Comparison of diagnosis for COVID-19 pneumonia based on RT-PCR results for cases with COVID-19 pneumonia on CT between ML-based CAST software and consensus evaluation or individual investigators’ evaluations

Groups	Method	SE (%)	SP (%)	AC (%)	*p* value for SE	*p* value for SP	*p* value for AC
Overall cases	ML-based software for CAST	97.4	16.7	82.1	–	–	–
		(75/77)	(3/18)	(78/95)			
	Final investigators’ evaluation	100	0	81.1	0.48	0.25	1
		(77/77)	(0/18)	(77/95)			
	Investigator A’s evaluation	90.9	55.6	84.2	0.18	0.07	0.82
		(70/77)	(10/18)	(80/95)			
	Investigator B’s evaluation	100	0	81.1	0.48	0.25	1
		(77/77)	(0/18)	(77/95)			
	Investigator C’s evaluation	98.7	27.8	85.3	1	0.68	0.51
		(76/77)	(5/18)	(81/95)			
Institution A cases	ML-based software for CAST	97.4	0	92.7	–	–	–
		(38/39)	(0/2)	(38/41)			
	Final investigators’ evaluation	100	0	95.1	1	N/A	1
		(39/39)	(0/2)	(39/41)			
	Investigator A’s evaluation	92.3	100	92.7	0.62	0.48	1
		(36/39)	(2/2)	(38/41)			
	Investigator B’s evaluation	100	0	95.1	1	N/A	1
		(39/39)	(0/2)	(39/41)			
	Investigator C’s evaluation	97.4	0	92.7	1	N/A	1
		(38/39)	(0/2)	(38/41)			
Institution B cases	ML-based software for CAST	97.4	18.8	74.1	–	–	–
		(37/38)	(3/16)	(40/54)			
	Final investigators’ evaluation	100	0	70.4	1	0.25	0.62
		(38/38)	(0/16)	(38/54)			
	Investigator A’s evaluation	89.5	50	77.8	0.37	0.18	0.79
		(34/38)	(8/16)	(42/54)			
	Investigator B’s evaluation	100	0	70.4	1	0.25	0.62
		(38/38)	(0/16)	(38/54)			
	Investigator C’s evaluation	100	31.3	79.6	1	0.68	0.45
		(38/38)	(5/16)	(43/54)			

*SE* sensitivity, *SP* specificity, *AC* accuracy

As for diagnosis for COVID-19 pneumonia based on RT-PCR results for all cases, specificity (SP) and accuracy (AC) of ML-based CAST software were significantly lower than those of consensus evaluation (SP: *p* < 0.0001, AC: *p* < 0.0001), investigator A (SP: *p* < 0.0001, AC: *p* < 0.0001) and investigator C (SP: *p* < 0.0001, AC: *p* < 0.0001). For institution A cases, SP of ML-based CAST software was significantly lower than that of consensus evaluation (*p* = 0.01), investigator A (*p* = 0.004) and investigator C (*p* = 0.01). For institution B cases, SP and AC of ML-based CAST software were significantly lower than those of consensus evaluation (SP: *p* = 0.0002, AC: *p* = 0.0001), investigator A (SP: *p* < 0.0001, AC: *p* = 0.0001) and investigator C (SP: *p* < 0.0001, AC: *p* < 0.0001).

A comparison of diagnosis for COVID-19 pneumonia based on RT-PCR results for cases with COVID-19 pneumonia findings on CT showed no significant differences between SE, SP and AC of diagnoses using ML-based CAST software and those using consensus evaluation as well as between those of each investigator for all cases (*p* > 0.05) and either institution (A: *p* > 0.05, B: *p* > 0.05).

Results of inter-rater agreement for each radiological finding among all investigators are shown in Table 6. Inter-rater agreements among all investigators were rated as “moderate” to “substantial” (0.55 ≤ Fleiss’s *κ* ≤ 0.8, *p* =  < 0.0001).

**Table 6:** Inter-rater agreement among all investigators for each radiological finding

Lung structure	Fleiss’ kappa value	*p* value
Consolidation	0.8	< 0.0001
Emphysema	0.64	< 0.0001
Ground-glass opacity	0.71	< 0.0001
Honeycombing	0.76	< 0.0001
Nodular lesion	0.73	< 0.0001
Reticulation	0.55	< 0.0001

Results of accuracy of evaluations for all radiological findings between the ML-based CT texture analysis using CAST software and each investigator are shown in Table 7. Comparison of accuracy of agreement for each radiological finding evaluation between ML-based CT texture analysis using CAST software and by each investigator showed that emphysema evaluation by investigator A was significantly lower than that by investigators B (*p* = 0.0009) and C (*p* = 0.0009).

**Table 7:** Comparison of accuracy for all radiological finding evaluations between ML-based CT texture analysis using CAST software and individual investigators’ evaluations

Lung structure	Investigator	Accuracy (%)	*p* value for comparison with investigator B	*p* value for comparison with investigator C
Consolidation	A	98.7	0.48	0.48
		(154/156)		
	B	100	–	N/A
		(156/156)		
	C	100	–	–
		(156/156)		
Emphysema	A	91.7	0.0009	0.0009
		(143/156)		
	B	100	–	N/A
		(156/156)		
	C	100	–	–
		(156/156)		
GGO	A	99.4	1	1
		(155/156)		
	B	100	–	1
		(156/156)		
	C	99.4	–	–
		(155/156)		
Honeycombing	A	96.2	0.13	0.13
		(150/156)		
	B	99.4	–	1
		(155/156)		
	C	99.4	–	–
		(155/156)		
Nodular lesion	A	98.7	0.48	0.48
		(154/156)		
	B	100	–	N/A
		(156/156)		
	C	100	–	–
		(156/156)		
Reticulation	A	99.4	1	1
		(155/156)		
	B	100	–	N/A
		(156/156)		
	C	100	–	–
		(156/156)		

*GGO* ground-glass opacity

## Discussion

Our results indicated that sensitivity of the newly developed ML-based CAST software had no significant difference with that of final evaluation by all investigators as well as each investigator’s evaluation in all cases, even though specificity and accuracy of ML-based CAST software were significantly lower than those of final investigators’ evaluation as well as investigator A’s and C’s evaluations. On the other hand, sensitivity, specificity, and accuracy of the software used for evaluation of patients with COVID-19 pneumonia on CT showed no significant differences with those of evaluations by the consensus evaluation nor by each investigator. Therefore, this software may be capable of functioning as a substitute for board-certified chest radiologists, especially in patients with COVID-19 pneumonia on CT. Moreover, accuracy for evaluations of all radiological findings was over 90%. This study is the first to report the findings for the performance of ML-based CAST software for management of COVID-19 pneumonia detected on CT using real-world data.

Agreements for diagnosis of COVID-19 pneumonia between CAST software and final evaluation or each investigator’s evaluation as well as inter-rater agreements for diagnosis of COVID-19 pneumonia among the three investigators, were rated as “moderate” in all cases [26–29]. Moreover, agreements for cases from institutions in a prefecture without collapse of the medical system were assessed as significantly moderate or substantial, although those for cases from institution in a prefecture with collapse of the medical system were slight or fair with or without statistical significance [26–29]. Therefore, collapse of the medical system is considered an important factor of influencing agreements between diagnosis based on the software and on the final evaluation as well as evaluation by each investigator [26–29].

For diagnosis of COVID-19 pneumonia based on RT-PCR results in all cases and cases from institutions with and without collapse of the medical system, specificity or accuracy of ML-based CAST software diagnoses were significantly lower than those of consensus evaluation, investigator A and investigator C. However, in cases with COVID-19 pneumonia detected on CT, there were no significant differences in diagnostic performance between ML-based CAST software and consensus evaluation as well as by each investigator. Moreover, there were no significant differences in diagnostic performance between ML-based CAST software and consensus evaluation as well as by each investigator, regardless of whether cases were obtained from institutions with or without collapse of the medical system. Therefore, our results indicate that ML-based CAST software could play as a substitute or in a complementary role for chest expert radiologists, when a given patient showed some radiological findings related to COVID-19 pneumonia. Therefore, this CAST software can provide a second opinion regarding COVID-19 pneumonia probability classification on CT as valid as the opinion of experienced chest radiologists and may, thus, make it possible to triage suspected COVID-19 pneumonia patients in routine clinical practice.

Comparisons of the diagnostic accuracy of each radiological finding evaluation on ML-based CAST software with that of three chest radiologists with equal to or more than 8-year experience showed that inter-rater agreement was “substantial” for all radiological findings except reticulation, which was rated “moderate”. Moreover, the diagnostic accuracy of all radiological findings may be considered relatively sufficient. Therefore, CT texture analysis results obtained in routine clinical practice with this CAST software can be considered highly acceptable, as highly as that of chest radiologists.

There are several limitations to this study. First, we used ML-based CAST software based on machine learning for evaluating radiological findings in accordance with the Fleischner Society Glossary and assessing CT findings for COVID-19 patients. However, this software was based on previously published machine learning software with proven capability to serve as a second reader to support expert radiologists and improve their intra- and inter-observer agreements on CT evaluations for various pulmonary parenchymal diseases. In addition, it reportedly has the capability to evaluate disease severity and predict therapeutic outcome using thin-section CT for COVID-19 patients [23, 24]. However, the study population and readers for our study were different from those for previous studies, and the performance level of the software used in our study was slightly lower than that reported for previously published results. Second, this was a retrospective study, and the training and validation data were obtained from two institutions and only a few CT systems from only one CT vendor were used. These facts might constitute one of the biases affecting this software’s performance. Third, all CT data for this study was obtained from Canon Medical Systems and analyzed with the CAST software provided by Canon Medical Systems, and not with additional software provided by other vendors or developed by other academics [30–33]. All CT data were obtained at two institutions, which use different CT protocols with various automatic exposure control systems, radiation doses, reconstruction algorithms, section thicknesses, etc. Although previous study analyzed by same software were assessed COVID-19 pneumonia patients with CT images obtained various CT vendors and different CT protocols [23], the above-mentioned issues were also considered as study results in this study. Fourth, COVID-19 infections are currently on the decline, and analysis of CT findings of COVID-19 pneumonia prior to the omicron is currently less clinical value in this time point. Moreover, although CT disease severity score for COVID-19 pneumonia was assessed in this study, severity of the COVID-19 patients was not directly assessed or predicted from CT findings or others in this study. In addition, we compared capability for diagnostic accuracy of our CAST software with expert radiologists but did not compare it with general clinicians. Furthermore, many Japanese people have already been vaccinated for COVID-19 and less people currently demonstrate ‘typical CT findings’ of COVID-19 pneumonia. Therefore, these facts were considered as biases in this study, and above-mentioned limitations or differences may have impacted our study results, especially quantitatively.

In conclusion, this multi-center study shows evaluations by CAST can be considered at least as valid as by chest expert radiologists for COVID-19 pneumonia triage with accurate radiological finding evaluations. This software, thus, may be able to play as complementary a role for management of suspected COVID-19 pneumonia patients as the RT-PCR test in routine clinical practice.

## Abbreviations

AC: Accuracy
CAST: Computer-aided simple triage
CT: Computed tomography
COVID-19: Coronavirus disease 2019
CRO: Clinical research organization
CTDI_vol_: Estimated volume computed tomography dose index
DLP: Dose length product
GGO: Ground-glass opacity
ML: Machine learning
PCR: Polymerase chain reaction
RT-PCR: Reverse transcription polymerase chain reaction
SARS-CoV-2: Severe acute respiratory syndrome coronavirus-2
SE: Sensitivity
SP: Specificity

## References

[1] WHO Coronavirus (COVID-19) Dashboard. Web site. https://covid19.who.int. Accessed 12 Apr 2023.

[2] World Health Organization. Novel Coronavirus (2019-nCoV): situation report, 1. World Health Organization. 2020. https://apps.who.int/iris/handle/10665/330760. Accessed 18 Dec 2022.

[3] Shi H, Han X, Jiang N (2020). Radiological findings from 81 patients with COVID-19 pneumonia in Wuhan, China: a descriptive study. Lancet Infect Dis.

[4] Ai T, Yang Z, Hou H (2020). Correlation of chest CT and RT-PCR testing for coronavirus disease 2019 (COVID-19) in China: a report of 1014 cases. Radiology.

[5] Xie X, Zhong Z, Zhao W, Zheng C, Wang F, Liu J (2020). Chest CT for typical coronavirus disease 2019 (COVID-19) pneumonia: relationship to negative RT-PCR testing. Radiology.

[6] Fang Y, Zhang H, Xie J (2020). Sensitivity of chest CT for COVID-19: comparison to RT-PCR. Radiology.

[7] Simpson S, Kay FU, Abbara S (2020). Radiological Society of North America expert consensus document on reporting chest CT findings related to COVID-19: endorsed by the Society of Thoracic Radiology, the American College of Radiology, and RSNA. Radiol Cardiothorac Imaging.

[8] Simpson S, Kay FU, Abbara S, Radiological Society of North America Expert Consensus Statement on Reporting Chest CT Findings Related to COVID-19 (2020). Endorsed by the society of thoracic radiology, the American College of Radiology, and RSNA—secondary publication. J Thorac Imaging.

[9] Prokop M, van Everdingen W, van Rees Vellinga T, COVID-19 Standardized Reporting Working Group of the Dutch Radiological Society (2020). CO-RADS: a categorical CT assessment scheme for patients suspected of having COVID-19-definition and evaluation. Radiology.

[10] British Society of Thoracic Imaging Web site. BSTI COVID-19 guidance for the reporting radiologist version 2.0. https://www.bsti.org.uk/standards-clinical-guidelines/clinical-guidelines/bsti-covid-19-guidance-for-the-reporting-radiologist/. Accessed 18 Mar 2023.

[11] Salehi S, Abedi A, Balakrishnan S, Gholamrezanezhad A (2020). Coronavirus disease 2019 (COVID-19) imaging reporting and data system (COVID-RADS) and common lexicon: a proposal based on the imaging data of 37 studies. Eur Radiol.

[12] Gezer NS, Ergan B, Barış MM (2020). COVID-19 S: a new proposal for diagnosis and structured reporting of COVID-19 on computed tomography imaging. Diagn Interv Radiol.

[13] Hare SS, Rodrigues JCL, Nair A (2020). The continuing evolution of COVID-19 imaging pathways in the UK: a British Society of Thoracic Imaging expert reference group update. Clin Radiol.

[14] Litmanovich DE, Chung M, Kirkbride RR, Kicska G, Kanne JP (2020). Review of chest radiograph findings of COVID-19 pneumonia and suggested reporting language. J Thorac Imaging.

[15] O’Neill SB, Byrne D, Müller NL (2021). Radiological Society of North America (RSNA) expert consensus statement related to chest CT findings in COVID-19 versus CO-RADS: comparison of reporting system performance among chest radiologists and end-user preference. Can Assoc Radiol J.

[16] Bellini D, Panvini N, Rengo M (2021). Diagnostic accuracy and interobserver variability of CO-RADS in patients with suspected coronavirus disease-2019: a multireader validation study. Eur Radiol.

[17] Hare SS, Tavare AN, Dattani V (2020). Validation of the British Society of Thoracic Imaging guidelines for COVID-19 chest radiograph reporting. Clin Radiol.

[18] Mondal MRH, Bharati S, Podder P (2021). Diagnosis of COVID-19 using machine learning and deep learning: a review. Curr Med Imaging.

[19] Islam MM, Poly TN, Walther BA (2020). Clinical characteristics and neonatal outcomes of pregnant patients with COVID-19: a systematic review. Front Med (Lausanne).

[20] Jia LL, Zhao JX, Pan NN (2022). Artificial intelligence model on chest imaging to diagnose COVID-19 and other pneumonias: a systematic review and meta-analysis. Eur J Radiol Open.

[21] Hansell DM, Bankier AA, MacMahon H, McLoud TC, Müller NL, Remy J (2008). Fleischner Society: glossary of terms for thoracic imaging. Radiology.

[22] Ohno Y, Aoyagi K, Takenaka D (2021). Machine learning for lung CT texture analysis: Improvement of inter-observer agreement for radiological finding classification in patients with pulmonary diseases. Eur J Radiol.

[23] Ohno Y, Aoyagi K, Arakita K (2022). Newly developed artificial intelligence algorithm for COVID-19 pneumonia: utility of quantitative CT texture analysis for prediction of favipiravir treatment effect. Jpn J Radiol.

[24] Ohno Y, Aoyagi K, Takenaka D (2022). Machine learning for lung texture analysis on thin-section CT: capability for assessments of disease severity and therapeutic effect for connective tissue disease patients in comparison with expert panel evaluations. Acta Radiol.

[25] Pan F, Ye T, Sun P (2020). Time course of lung changes at chest CT during recovery from coronavirus disease 2019 (COVID-19). Radiology.

[26] Svanholm H, Starklint H, Gundersen HJ, Fabricius J, Barlebo H, Olsen S (1989). Reproducibility of histomorphologic diagnoses with special reference to the kappa statistic. APMIS.

[27] Landis JR, Koch GG (1977). The measurement of observer agreement for categorical data. Biometrics.

[28] Fleiss JL (1981). Statistical methods for rates and proportions.

[29] Mucci B, Murray H, Downie A, Osborne K (2013). Interrater variation in scoring radiological discrepancies. Br J Radiol.

[30] Feng Z, Shen H, Gao K (2021). Machine learning based on clinical characteristics and chest CT quantitative measurements for prediction of adverse clinical outcomes in hospitalized patients with COVID-19. Eur Radiol.

[31] Mortani Barbosa EJ, Georgescu B, Chaganti S, Aleman GB (2021). Machine learning automatically detects COVID-19 using chest CTs in a large multicenter cohort. Eur Radiol.

[32] Hurt B, Rubel MA, Masutani EM (2022). Radiologist-supervised transfer learning: improving radiographic localization of pneumonia and prognostication of patients with COVID-19. J Thorac Imaging.

[33] Jungmann F, Müller L, Hahn F (2022). Commercial AI solutions in detecting COVID-19 pneumonia in chest CT: not yet ready for clinical implementation?. Eur Radiol.

